# PGC-1α alternative promoter (Exon 1b) controls augmentation of total PGC-1α gene expression in response to cold water immersion and low glycogen availability

**DOI:** 10.1007/s00421-020-04467-6

**Published:** 2020-08-25

**Authors:** R. Allan, J. P. Morton, G. L. Close, B. Drust, W. Gregson, A. P. Sharples

**Affiliations:** 1 Research Institute for Sport and Exercise Sciences, Liverpool John Moores University, Liverpool, UK; 2 Division of Sport, Exercise and Nutritional Sciences, University of Central Lancashire, Preston, UK; 3 Institute of Physical Performance, Norwegian School of Sport Sciences, Oslo, Norway

**Keywords:** CWI, PGC-1α, Exon

## Abstract

This investigation sought to determine whether post-exercise cold water immersion and low glycogen availability, separately and in combination, would preferentially activate either the Exon 1a or Exon 1b Peroxisome proliferator-activated receptor gamma coactivator 1-alpha (PGC-1α) promoter. Through a reanalysis of sample design, we identified that the systemic cold-induced augmentation of total PGC-1α gene expression observed previously (Allan et al. in J Appl Physiol 123(2):451–459, 2017) was largely a result of increased expression from the alternative promoter (Exon 1b), rather than canonical promoter (Exon 1a). Low glycogen availability in combination with local cooling of the muscle (Allan et al. in Physiol Rep 7(11):e14082, 2019) demonstrated that PGC-1α alternative promoter (Exon 1b) expression continued to rise at 3 h post-exercise in all conditions; whilst, expression from the canonical promoter (Exon 1a) decreased between the same time points (post-exercise–3 h post-exercise). Importantly, this increase in PGC-1α Exon 1b expression was reduced compared to the response of low glycogen or cold water immersion alone, suggesting that the combination of prior low glycogen and CWI post-exercise impaired the response in gene expression versus these conditions individually. Data herein emphasise the influence of post-exercise cooling and low glycogen availability on Exon-specific control of total PGC-1 α gene expression and highlight the need for future research to assess Exon-specific regulation of PGC-1α.

## Introduction

Recently, it has been shown that skeletal muscle expresses several different transcript variants that produce different protein isoforms of the transcriptional co-activator peroxisome proliferator-activated receptor gamma co-activator 1-alpha (PGC-1α) (reviewed in Popov et al. 2015a). To date, more than eight alternative protein isoforms have been described in human skeletal muscle, where the gene expression of these variants is thought to occur via two alternate promoter regions on the same gene (Popov et al. 2015a; Martinez-Redondo et al. 2016). The proximal (canonical) PGC-1α gene promoter (Exon 1a) is constitutively expressed at rest compared to the alternative promoter (Exon 1b), whilst the alternative promoter (Exon 1b) is more responsive following exercise (Martinez-Redondo et al. 2015; Popov et al. 2015b). In humans, gene expression of Exon 1c is not highly expressed post-exercise with levels reported to be very low to negligible (Popov et al. 2015b).

Whilst evidence exists that supports exercise regulation of PGC-1α via a program of expression of distinct transcript variants, the contribution of post-exercise cold water immersion (CWI) or prior low muscle glycogen availability in the activation of PGC-1α via either the canonical (Exon 1a) or alternative (Exon 1b) promoter has not been determined. PGC-1α gene expression driven by exercise, cold and low glycogen stimuli is thought to be regulated by AMP-activated protein kinase (AMPK) (Norrbom et al. 2011; Wen et al. 2014) or β-adrenergic pathways (Chinsomboon et al. 2009; Tadaishi et al. 2011). Importantly, it has been suggested that Exon 1a and 1b are AMPK activated, whilst Exon 1b is also susceptible to β-adrenergic stimulation (Popov et al. 2015a). Recently, Brandt et al. (2017) showed increased promoter-specific gene expression in mice following a single injection of clenbuterol (a powerful β-adrenergic agonist), suggesting an important role for adrenergic signalling. Cold water immersion has been consistently shown to raise post-exercise plasma normetanephrine concentrations to a greater extent than non-immersed conditions (Joo et al. 2016; Allan et al. 2017), with post-exercise CWI augmenting the exercise-induced gene expression of total PGC-1α vs. a non-immersed control condition (Ihsan et al. 2014; Joo et al. 2016; Allan et al. 2017). Therefore, of particular interest is whether the individual impact of exercise, cold stress and low glycogen would preferentially activate either the Exon 1a or Exon 1b PGC-1α promoter, and whether the combined impact of these stimuli would further enhance PGC-1α gene expression. Such mechanistic information would allow greater support for the application of post-exercise CWI in an applied setting, whilst going some way to assist the correct use and timing of such modalities.

Thus, the aim of the present study was to assess the impact of post-exercise cooling and prior low glycogen availability on PGC-1α promoter-specific mRNA expression. To this end, we completed new analysis on the same RNA derived from the recently published work completed in our lab in which both post-exercise local and systemic cooling of human skeletal muscle tissue was undertaken (Allan et al. 2017) and the impact of combined post-exercise cooling and low glycogen availability within skeletal muscle (Allan et al. 2019) was assessed. This allowed us to assess the post-exercise PGC-1α promoter variant response, with low glycogen availability and post-exercise cooling alone, as well as combined. It was hypothesised that cold water immersion and prior low glycogen would increase the exercise-induced PGC-1α alternative (Exon 1b), yet based on recent evidence (Allan et al. 2019), the impact of combining these stressors was expected not to be advantageous.

## Materials and methods

### Ethical approval

All participants gave written informed consent to participate after details and procedures of studies had been fully explained. All participants had no history of neurological disease or musculoskeletal abnormality and none were under any pharmacological treatment during the course of the study. All procedures performed in the study were approved by Liverpool John Moores University Ethics Committee and in accordance with the 1964 Helsinki declaration and its later amendments.

## Study overview

**Fig. 1:**
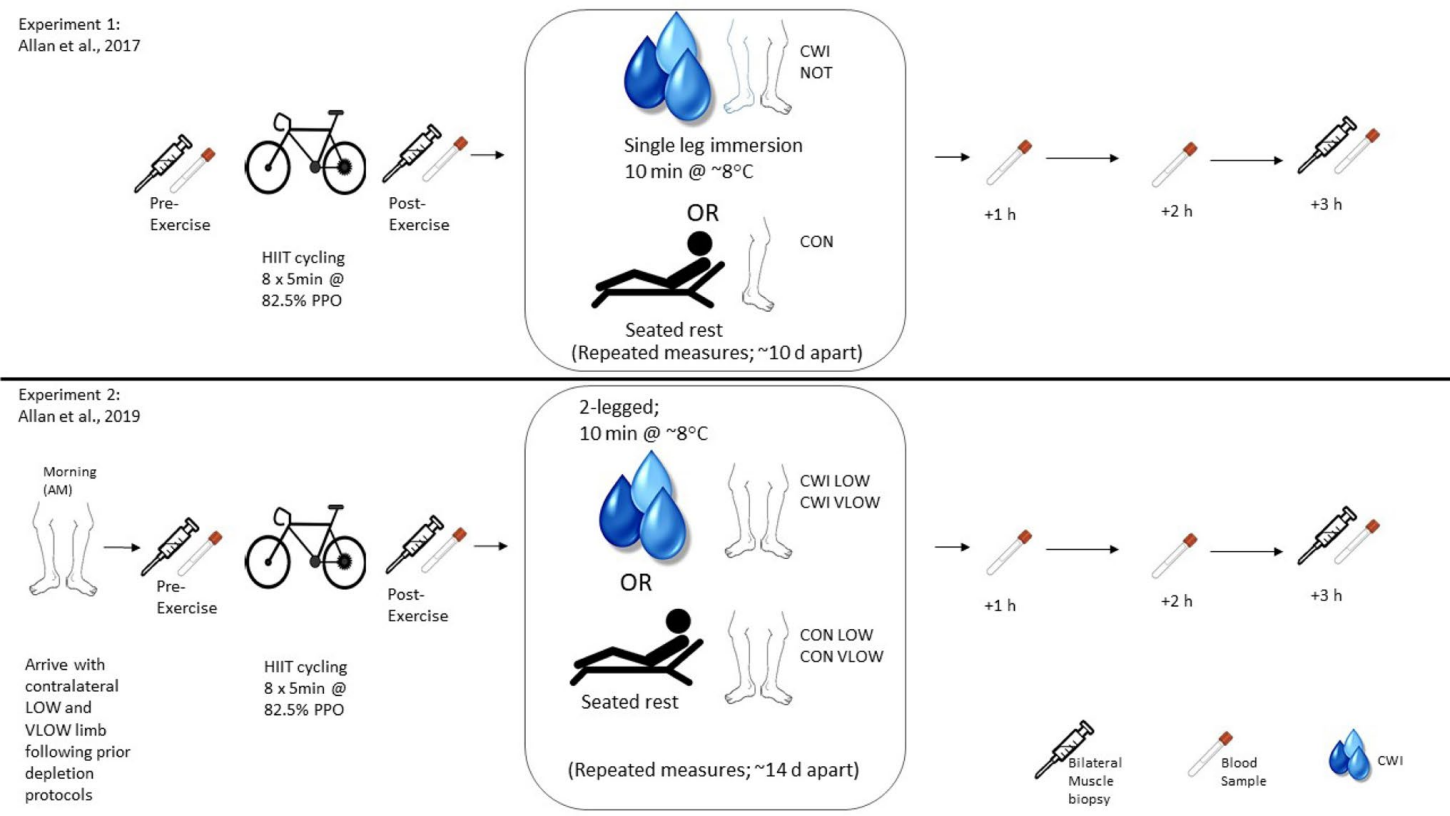
Overview of the experimental protocol used in each trial. Whilst blood samples were collected in the original studies no new data herein is derived from blood.  *HIIT* high-intensity intermittent exercise; *CON* control condition/limb; *NOT* non-immersed limb; *CWI* cold water immersion condition/limb; *PPO* peak power output; *LOW* low CHO limb; *VLOW* very low CHO limb

During this investigation, we undertook new analysis of existing RNA samples derived from previous work and methods are as previously published and shall be referred to herein as Experiment 1 (Allan et al. 2017) and Experiment 2 (Allan et al. 2019) (see Fig. 1 for experimental designs).

### Experimental design

For analysis of samples derived from Experiment 1 (Allan et al. 2017), participant data and experimental protocol have already been described; however, briefly, ten recreationally active healthy males (age 26 ± 4 years; body mass 79.29 ± 6.73 kg; height 180 ± 5 cm; V̇O_2peak_ 51.46 ± 9.07 mL·kg^−1^·min^−1^ peak power output (PPO) 265.2 ± 38.33 W; mean ± SD) participated in this study. Following pre-exercise assessments, participants completed a high-intensity intermittent cycling protocol (Lode Excalibur Sport, Lode, Netherlands) consisting of 8 × 5 min bouts at 82.5% PPO separated by 1-min rest followed by either one-legged CWI (CWI: 10 min at 8 ± 1 °C) or a control condition (CON; seated rest at room temperature).

In Experiment 2 (Allan et al. 2019), the impact of combined post-exercise local cooling in combination with starting low glycogen content within skeletal muscle was assessed. Nine recreationally active healthy males (age 22 ± 3 years.; body mass 74.18 ± 7.88 kg; height 180.50 ± 6.60 cm; peak power output (PPO) 271.8 ± 25.8 W; mean ± SD) participated in the study. Following a series of single- and double-leg glycogen-depleting protocols in the 2 days prior to the experimental day, participants attended the lab with one limb higher in glycogen availability than the other (LOW ~ 190 mmol kg^−1^dw and VLOW ~ 85 mmol kg^−1^dw). Following pre-exercise assessments, participants completed a high-intensity intermittent cycling protocol (Lode Excalibur Sport, Lode, Netherlands) consisting of 8 × 5 min bouts at 82.5% PPO separated by 1-min rest followed by either two-legged CWI (CWI: 10 min at 8 ± 1 °C) or a control condition (CON; seated rest).

“For both Experiments”, participants were instructed to refrain from exercise, alcohol and caffeine 48 h prior to attending the lab. Participants were lowered and raised into and out of the water to the level of the iliac crest using an electronic hoist system, after which they remained seated in a semi-reclined position in normal laboratory temperatures (~ 21 °C) until 3 h post-exercise. Muscle biopsies were obtained at pre-exercise, immediately post-exercise and 3 h post-exercise.

### Muscle analysis

Muscle biopsies were obtained from the vastus lateralis muscle under local anaesthesia (0.5% marcaine) using a Pro-Mag 2.2 biopsy gun (MD-TECH, Manan Medical Products, Northbrook, IL, USA). Each incision was anaesthetised individually and occurred at a distance of 2–3 cm from the previous incision. Once obtained, samples (~ 30–50 mg of tissue) were immediately frozen in liquid nitrogen and stored at − 80 °C for later analysis.

#### Real-time quantitative RT-PCR

RNA extraction and quantitative real-time PCR was conducted as previously described (Allan et al. 2017, 2019). Primer sequences (Table 1) were identified using Gene (NCBI, https://www.ncbi.nlm.nih.gov/gene) and designed using Primer-BLAST (NCBI, https://www.ncbi.nlm.nih.gov/tools/primer-blast/). Sequence homology searches ensured specificity and that all primers had no potential unintended targets. All primers were between 20 and 25 bp and amplified a product of between 127 and 168 bp. Primers were purchased from Sigma (Suffolk, UK). For exon-specific analysis, following the initial screening of suitable reference genes, GAPDH showed the most stable threshold cycle (C_T_) values across all RT-PCR runs and subjects regardless of experimental condition (27.02 ± 1.96 C_T_; 7% coefficient of variation) and was selected as the reference gene in all RT-PCR assays. The average PCR efficiency was 90%, and variation for all target genes including the reference gene was low at 2.8%. The relative gene expression levels were calculated using the comparative C_T_ (ΔΔC_T_) equation (Schmittgen and Livak 2008), where the relative expression was calculated as 2^–ΔΔCT^ and where C_T_ represents the threshold cycle. mRNA expression for all target genes was calculated relative to the reference gene (GAPDH; subject’s own sample reference) within the same subject and condition, and to a calibrator of pre-exercise.

**Table 1:** Primer sequences used for real-time PCR

Gene	Forward primer	Reverse primer	Product Length (base pairs)	Location
**GAPDH** NM_002046.5	AAGACCTTGGGCTGGGACTG	TGGCTCGGCTGGCGAC	168	6–173
**Total PGC-1_α** NM_013261.3	TGCTAAACGACTCCGAGAA	TGCAAAGTTCCCTCTCTGCT	67	
**Exon 1a** NM_013261.4 Recently updated to NM_013261.5	ATGGAGTGACATCGAGTGTGCT	GAGTCCACCCAGAAAGCTGT	127	159–285
**Exon 1b** XM_011513766.1 XM_005248132.1	CTATGGATTCAATTTTGAAATGTGC	CTGATTGGTCACTGCACCAC	153	77–229

*Exon 1a* PGC-1α Exon 1a; *Exon 1b* PGC-1α Exon 1b

### Statistical analysis

#### Experiment 1

A two-factor (3 conditions x time), within-participants, general linear model was used to evaluate the effect of time (preexercise vs. post-exercise) with shared baseline data used for NOT and CWI (Statistical package for the Social Sciences version 21.0; SPSS, Chicago, IL). A two-factor (3 conditions x time), within-participants, general linear model was subsequently used to evaluate the influence of the cooling intervention following exercise and the 3-h post exercise period. 

#### Experiment 2

A two-factor (4 condition x time) within-participant’s general linear model for condition (Low glycogen control (CON LOW), Very Low glycogen control (CON VLOW), Low glycogen CWI (CWI LOW), Very Low glycogen CWI (CWI VLOW)) and time (Pre, Post, 3 h) was used to evaluate the influence of low glycogen availability and CWI following exercise and the 3-h post-exercise period.

“For both experiments”, the main effects for condition and time were followed up via the use of planned least significant difference (LSD) multiple contrasts. Where a significant condition-by-ime interaction was observed, the post-exercise to 3-h post-exercise change scores were calculated and compared across conditions using LSD multiple contrasts. The ES magnitude was classified as trivial (< 0.2), small (> 0.2–0.6), moderate (> 0.6–1.2), large (> 1.2–2.0) and very large (> 2.0–4.0) (Hopkins et al. 2009). Data are means ± SD unless otherwise stated. The α level for evaluation of statistical significance was set at *P* < 0.05.

## Results

### Experiment 1

**Fig. 2:**
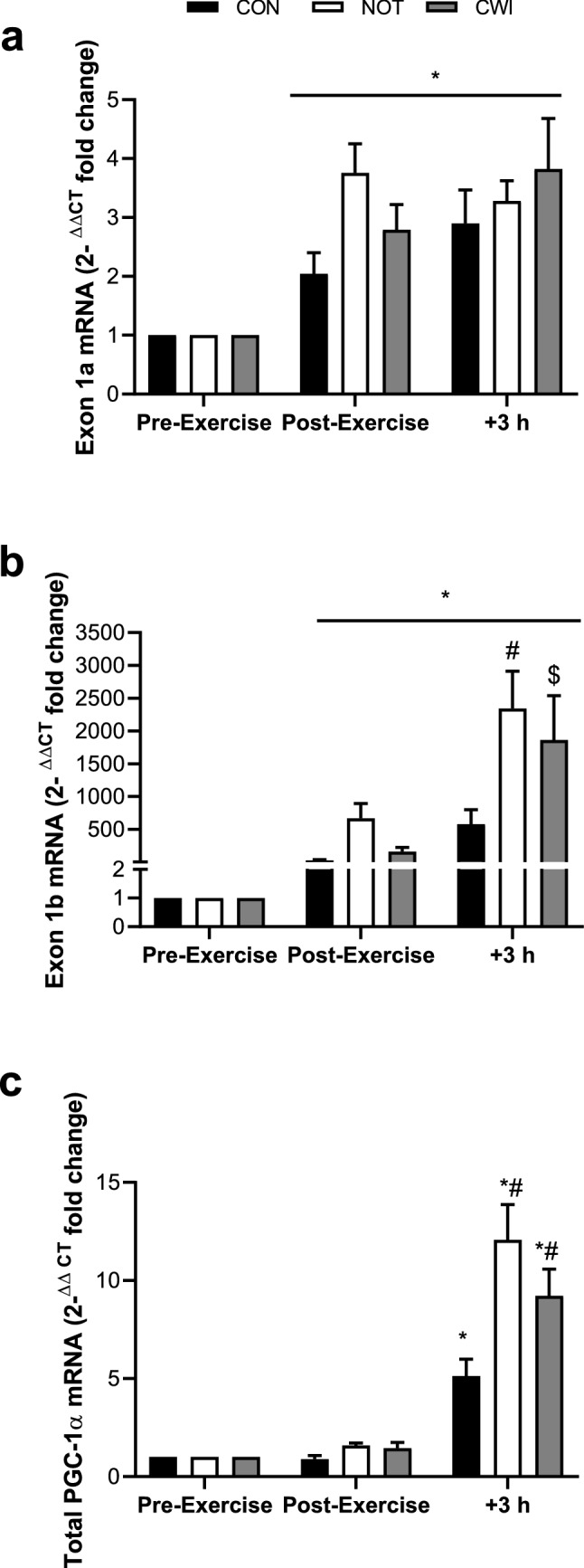
PGC-1α mRNA and specific to Exon 1a (**a**) and Exon 1b (**b**) ^ΔΔCT^ fold change in expression value with the calibrator as pre-exercise and the reference gene as GAPDH. Values are mean ± SEM. *Significantly different from pre-exercise (*P* < 0.05). #Significantly different from CON (*P* < 0.05). $ (*P* = 0.07) vs CON. + 3 h = 3 h post-exercise. Total PGC-1α mRNA (**c**) redrawn from Allan et al. (J Appl Physiol. 2017; 123 (2): 451–459) as mean ± SEM

Exercise significantly increased the expression of Exon 1a mRNA ~ two–four fold from Pre (Pre–Post-Exercise: *P* < 0.001, ES 2.50 Very Large, Fig. 2). In the 3-h post-exercise recovery period, no change in level of expression was seen (*P* = 0.202, ES 0.27 Small) between conditions (*P* = 0.146, ES 0.74 Moderate). The change over time was significant (*P* = 0.05). Exercise also significantly increased the expression of Exon 1b mRNA (Pre–Post-Exercise: *P* = 0.016, ES 1.14 Large, Fig. 2). In the 3-h post-exercise recovery period, Exon 1b expression increased significantly (*P* = 0.007, ES 1.15 Large) between conditions (*P* = 0.011). Post hoc analysis showed NOT (2344-fold change from Pre, ES 1.12 Large) significantly increased Exon 1b gene expression compared to CON (579-fold change from Pre, *P* = 0.010) and CWI (1860-fold change from Pre, ES 0.61 Moderate) on average increased Exon 1b gene expression versus CON, an observation that approached statistical significance (*P* = 0.07). No significant change in Exon 1b gene expression between NOT and CWI (*P* = 0.185, ES 0.29 Small) was evident. No significant interaction effect was present (*P* = 0.092).

### Experiment 2

**Fig. 3:**
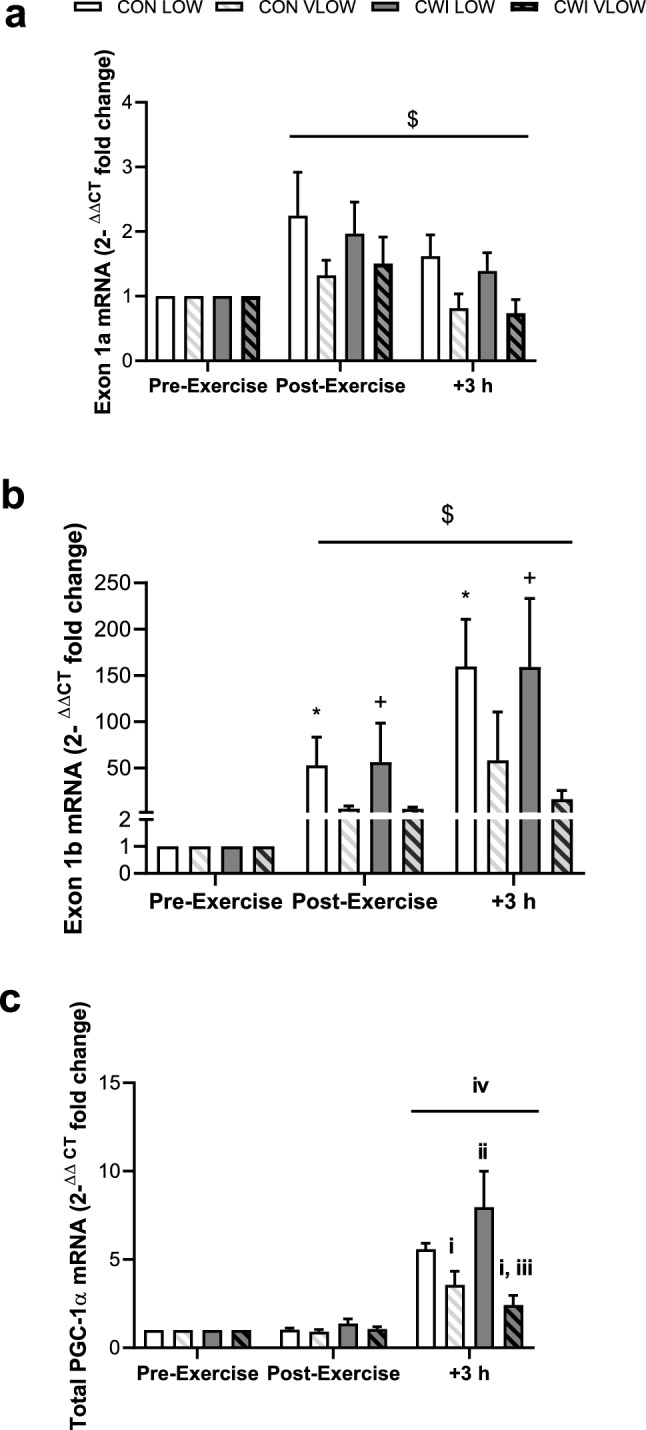
PGC-1α mRNA specific to Exon 1a (**a**) and Exon 1b (**b**) ^ΔΔCT^ fold change in expression with the calibrator as pre-exercise and the reference gene as GAPDH. Values are mean ± SEM. $ Trend towards increase from pre-exercise (**a** *P* = 0.065, **b** *P* = 0.057). *Significantly different from corresponding LOW limb (*P* = 0.025). + Different from corresponding LOW limb (*P* = 0.062). + 3 h = 3 h post-exercise. Total PGC-1α mRNA (**c**) redrawn from Allan et al. (Physiol Rep. 2019; 7(11): e14082) as mean ± SEM. ^i^Significantly less than CON LOW (*P* < 0.05), ^ii^ greater than CON VLOW (*P* = 0.05), ^iii^ Significantly less than CWI LOW (*P* = 0.019), ^iv^Significantly greater than pre- and post-exercise (*P* < 0.001)

Exercise evoked an increase in the expression of Exon 1a mRNA, peaking immediately post exercise (~ 1.5–twofold change from Pre, ES 1.07 Moderate) before decreasing towards pre-exercise values at 3 h post-exercise (~ 1–1.5-fold change from Pre, *P* = 0.065, ES 0.33 Small, Fig. 3). No statistical significance was present between conditions or for the change over time (*P* = 0.086 and 0.492, respectively). Exercise evoked an increase in the expression of Exon1b mRNA, with the greatest increases seen at 3 h post-exercise (CON LOW and CWI LOW ~ 159-fold change from Pre, CON VLOW ~ 58-fold change from Pre, CWI VLOW ~ 56-fold change from Pre, *P* = 0.057, ES 1.20 Moderate/Large). Significant differences were observed between conditions (*P* = 0.035, Fig. 3) with both LOW limbs having greater expression than their corresponding VLOW limb at 3 h post-exercise (CON *P* = 0.025, ES 0.67 Moderate; CWI *P* = 0.062, ES 1.14 Large). No difference was present in both VLOW and LOW limbs when the CWI intervention was applied (VLOW *P* = 0.398, ES 0.26 Small; LOW *P* = 0.966, ES 0.007 Trivial) suggesting that a cold-induced response in Exon 1b gene expression was not present. No significant interaction was seen (*P* = 0.111).

## Discussion

This is the first study to investigate the relative influence of post-exercise cooling as well as the impact of reduced glycogen availability alone, and in combination, on promoter-specific mRNA expression of the transcriptional co-activator PGC-1α. Experiment 1 provided novel results demonstrating PGC-1α mRNA expression from the alternative promoter (Exon 1b) mirrors the pattern of expression for total PGC-1α mRNA previously described (Allan et al. 2017) (Fig. 2). Thus, the contribution to total PGC-1α mRNA expression post-exercise is likely a result of changes occurring from the alternative promoter (Exon 1b). Moreover, a systemic response to CWI was seen in the alternative promoter (Exon 1b) with large (~ 2000) fold changes from pre-exercise in the immersed and contralateral non-immersed limbs, versus a non-immersed control (< 1000-fold change from Pre) suggesting the response occurs independent of tissue temperature.

When muscle glycogen availability is very low (Experiment 2) (< 150 mmol·kg^−1^dw; VLOW), the cold-induced augmented gene expression for total PGC-1α mRNA was abolished (Allan et al. 2019). New analysis of RNA samples herein focussing on the exon-specific response highlights VLOW glycogen limbs showing reduced mRNA expression via the alternative promoter (Exon 1b) versus the contralateral LOW glycogen limb, supporting the recent data indicating a glycogen threshold for adequate increases in gene expression to occur (Impey et al. 2018; Hearris et al. 2019). Moreover, a cold stimulus had no influence upon promoter-specific expression (Exon 1a and 1b) when glycogen concentration was low. Indeed, the combination of very low glycogen availability and post-exercise CWI showed the largest impaired response in gene expression of PGC-1α, irrespective of promoter region (Exon 1a and 1b). In Experiment 1, expression from the canonical promoter (Exon1a) showed a significant increase post-exercise in all limbs (two–fourfold change from Pre, *P* < 0.05). Larger fold changes were present in expression from the alternative promoter (Exon1b), with values ranging ~ 1000–2000-fold increases from pre-exercise values (*P* < 0.05). The sizeable difference in change of expression between the different promoter regions was likely due to the differential expression at pre-exercise (rest) and response to an exercise stimulus. Indeed, Exon 1a is constitutively expressed at higher levels basally (at rest) to a greater extent than Exon 1b, whilst Exon 1b is more responsive to an exercise stimulus (Martinez-Redondo et al. 2015; Popov et al. 2015b). In the present study, the pattern of response following exercise from the alternative (Exon 1b) promoter closely mirrored that of total PGC-1α gene expression seen in the same subjects previously (Allan et al. 2017), offering support to evidence showing the response of total PGC-1α mRNA to acute exercise is largely driven by the alternative promoter (Exon 1b) (Norrbom et al. 2011; Ydfors et al. 2013; Silvennoinen et al. 2015; Popov et al. 2015b). Additionally, new data herein to investigate the impact of commencing exercise with low glycogen availability combined with post-exercise cooling on the promoter-specific response (Experiment 2) show that PGC-1α alternative promoter (Exon 1b) expression continued to rise at 3 h post-exercise in all conditions, whilst expression from the canonical promoter (Exon 1a) decreased between the same time points (post-exercise–3 h post-exercise). This emphasises the role of the alternative promoter to the exercise-induced augmentation in total PGC-1α gene expression (Allan et al. 2017, 2019). Importantly, in Experiment 1, PGC-1α gene expression from the alternative promoter (Exon 1b) showed large increases in response to high-intensity intermittent cycling (CON, < 1000-fold change from Pre), which was augmented even further in limbs exposed to systemic cold stress (NOT and CWI, ~ 2000-fold change from Pre, *P* = 0.07 and < 0.05 vs. CON, respectively). Indeed, the same pattern of response was observed in total PGC-1α mRNA (Allan et al. 2017), and the present data suggest that the contribution to cold-induced total PGC-1α gene expression is also driven by the alternative (Exon1b), and not the canonical (Exon1a) promoter region. These data are the first to show such a promoter-specific response to cold in animal or human studies.

In conclusion, this is the first study to show the systemic cold-induced augmentation of total PGC-1α as seen previously (Allan et al. 2017) is largely a result of increased expression from the alternative promoter (Exon 1b), rather than canonical promoter (Exon 1a). Evidence herein further supports the notion that PGC-1α gene expression from the alternative promoter (Exon 1b) is more reactive to an exercise stimulus than the canonical promoter region; with Exon 1b demonstrating lower levels at baseline, but more responsive to cold and exercise so demonstrating large fold increases. Whereas, Exon 1a was expressed more highly at rest and was less responsive to exercise and the cold and so demonstrates smaller fold increases. Moreover, commencing exercise with extremely low glycogen concentrations seems to negate the expected increase in PGC-1α gene expression irrespective of promoter region (Exon 1a and b), supporting the recent glycogen threshold hypothesis (Impey et al. 2018; Hearris et al. 2019). These data also provides greater support to the knowledge that post-exercise cooling may benefit important molecular signals of mitochondrial biogenesis, allowing confidence in the fact that endurance adaptation may be augmented following such modalities. Future research should look to include assessment of PGC-1α promoter-specific gene expression to allow greater mechanistic understanding of this important transcriptional co-activator.
